# Effects of physical exercise on college students’ academic self-efficacy: the chain mediating role of future orientation and mental toughness

**DOI:** 10.3389/fpsyg.2025.1604725

**Published:** 2025-05-27

**Authors:** Yong Jiang, Yifeng Fu, Xinqiu Dong

**Affiliations:** ^1^Liaoning Normal University School of Physical Education, Dalian, China; ^2^Jilin University School of Physical Education, Changchun, China

**Keywords:** physical exercise, academic self-efficacy, future orientation, mental toughness, chain mediation

## Abstract

**Introduction:**

Academic self-efficacy affects students’ behavioral styles, efforts, and attitudes toward academic difficulties in the learning process. Physical education, as a core vehicle of education, and physical exercise, as an important way to promote physical and mental health, have special value for the development of academic self-efficacy. The study explored the effects of physical exercise on college students’ academic self-efficacy and the chain-mediated effects of future orientation and mental toughness.

**Methods:**

A total of 624 college students were surveyed using the physical exercise Rating Scale, Academic Self-Efficacy Scale, Future Orientation Scale, and Mental Toughness Scale, and the mediating role of Future Orientation and Mental Toughness was examined and analyzed by using Structural Equation Modeling and Bootstrap Method.

**Results:**

① physical exercise and academic self-efficacy were positively correlated, and the direct prediction of academic self-efficacy was significant; ② physical exercise can positively predict future orientation, future orientation can positively predict mental toughness and academic self-efficacy, and mental toughness can positively predict academic self-efficacy; ③ future orientation and mental toughness play a significant mediating role in the relationship between physical exercise and academic self-efficacy. The three pathways of mediating effects: physical exercise and academic self-efficacy, and mental toughness. Three pathways of mediating effects were found: physical exercise→future orientation→academic self-efficacy (Path 1), physical exercise→mental toughness→academic self-efficacy (Path 2), and physical exercise→future orientation→mental toughness→academic self-efficacy (Path 3).

## 1 Introduction

The World Health Organization (WHO) has released several sport and physical exercise-related documents aimed at promoting global health. One of them, Guidelines on Physical Exercise and Sedentary Behavior, provides physical exercise recommendations for different age groups, including children, adults, older adults, pregnant women, and people with chronic diseases and disabilities, emphasizing the importance of reducing sedentary behavior. For adolescents, physical exercise of moderate to vigorous intensity for at least 1 h per day is recommended (GBD 2015 Mortality and Causes of Death Collaborators, 2016; Hallal et al., 2012; Strain et al., 2024). “When the youth is strong, China is strong, and when sports is strong, China is strong.” College students are the future of the country and the hope of the nation, but the physical and mental health crisis of college students caused by insufficient physical exercise or lack of physical exercise has become a global problem. Especially in college, regular physical exercise can help students relieve stress, enhance self-confidence and adaptability, and thus have a positive effect on academic performance (Zhang and Min, 2022). The improvement of students’ academic self-efficacy is particularly important. Academic self-efficacy is a learner’s judgment of his or her ability to successfully achieve academic goals (Elias and Macdonald, 2007).

Future orientation and psychological resilience, as important concepts in positive psychology, play key roles in this mechanism of influence (Deng et al., 2023). Future orientation is the direction in which an individual’s thoughts and behaviors point to the future, foreshadowing the individual’s goal setting, expectations for the future, and this series of thinking and planning processes (Xiao et al., 2022). Mental toughness is a psychological trait that has gradually gained attention after the emergence of positive psychology, which refers to an individual’s flexibility to cope with setbacks and adversity, from which he or she maintains or quickly recovers his or her normal psychological function (Zhao et al., 2024). However, there is a lack of systematic research on how physical exercise affects college students’ academic self-efficacy through the chain path of future orientation and psychological toughness. This study takes physical exercise as the logical starting point of the study, analyzes the mechanism of its influence on academic self-efficacy, examines the role of future orientation and mental toughness, explores the indirect influence mechanism of physical exercise on college students’ academic self-efficacy, and reveals the deeper path of the effect of physical exercise on the psychological and academic development of college students, with the aim of promoting the all-around growth and the improvement of the comprehensive quality of college students. The purpose of this study is to promote the comprehensive growth and quality improvement of college students.

## 2 Theoretical basis and hypothesis

### 2.1 The effect of physical exercise on college students’ academic self-efficacy

Physical exercise refers to physical activities completed with a certain intensity, frequency and duration with the aim of benefiting physical and mental health (Pan et al., 2021). It is an active, healthy lifestyle that incorporates natural forces to exercise the body. It promotes both the physical and mental health of an individual, and will also change an individual’s habits and behaviors (Wei, 2023). Academic self-efficacy is the specific use of self-efficacy in the field of learning, refers to the judgment of students on their ability to successfully complete the learning task, is an individual’s subjective judgment and subjective feelings about controlling their own learning behaviors and learning ability (Bandura, 1986; Honicke et al., 2019). A study found that academic self-efficacy is a materialization of self-efficacy theory in the field of learning, and that physical exercise and self-efficacy can promote each other, and have a strong predictive effect on each other (Li, 2022; Qin et al., 2024). Physical exercise has a significant positive effect on college students’ academic self-efficacy (Shen, 2020; Sheng et al., 2019). That is, the higher the frequency, intensity and duration of participation in physical exercise, the higher the level of academic self-efficacy of college students. This is because the positive physical and psychological changes brought about by physical exercise can enable college students to assess their abilities and potentials more optimistically in academic situations, and to believe that they can effectively complete their learning tasks and achieve good academic results. For example, college students who regularly participate in physical exercise may show higher academic self-efficacy due to the fact that they exercise their willpower and concentration during exercise, and they are also able to devote their energy more firmly and believe that they are able to cope when facing complex academic research or exam revision, thus showing higher academic self-efficacy. Based on the above analysis, this study proposes Hypothesis H1: Physical exercise positively predicts college students’ academic self-efficacy.

### 2.2 Mediating effect of future orientation

It can be hypothesized that future orientation plays a mediating role between physical exercise and academic self-efficacy by combing through previous studies. Future Orientation embodies an individual’s vision, planning and sensitive attitude toward the future, and its dimensions cover future cognition, which is the clear expectation of academic goals; future positive emotion, which is the emotional experience that may arise in the process of goal achievement; and future will-action tendency, which is the specific behavioral strategy adopted to achieve the goal (Liu et al., 2010). Future orientation is a crucial part of self-development, as a future-oriented psychological structure, it can not only give important power to guide the individual’s cognitive activities in the present, but also promote the individual’s behavior in the present toward the future goals and serve them (Wang, 2011). It has been found that physical exercise can enhance students’ visions of the future and help them form a more positive future orientation (Huang, 2017). High future orientation can directly and positively correlate with academic self-efficacy, a tendency to set specific long-term goals and maintain goal-oriented behavior by breaking down tasks and reviewing them regularly (Li et al., 2017). Those who are clear about their future orientation have higher self-efficacy and are more proactive in participating in research projects to accumulate experience, while students with a strong tendency to take action make daily study plans to ensure that their goals are gradually realized (Fang, 2020), and the better they perform academically (Pu et al., 2023). Therefore, physical exercise can enhance college students’ physical health and psychological health and make them pay more attention to their future orientation, thus setting higher academic goals and improving their academic self-efficacy. Based on the above analysis, this study proposes the hypothesis H2: Future orientation has a mediating role between physical exercise and college students’ academic self-efficacy.

### 2.3 Mediating effects of mental toughness

Positive psychologist Luthans (2008) defines psychological resilience as “the existence of an individual’s potential to be tapped for rapid recovery from adversity, failure, positive practice, and increasing responsibility.” Mental toughness also known as psychological elasticity, psychological resilience or resistance, which belongs to the individual in the face of stress, frustration and trauma with a kind of ability or trait, is extremely critical to the positive psychological qualities (Ruiz, and Pedro, 2012). In the college student population, mental toughness is specifically manifested in coping with test anxiety, getting rid of academic setbacks, and regulating emotional stress. Research in the field of sports has found that physical exercise can positively predict the mental toughness of college students (Liu, 2020), and some studies have shown that moderate-intensity physical exercise can effectively enhance the mental toughness of college students (Hu, 2019); the mental toughness of college students who participate in moderate and above physical exercise is significantly better than that of those who participate in small-volume exercise (Song et al., 2014). Individuals with higher levels of mental toughness not only can handle setbacks appropriately and maintain emotional stability during academic setbacks, but also take the initiative to explore opportunities and challenges for their own growth, tend to focus on their own goals, and cope with challenges with ease, and have confidence in their own abilities (Mcgeown et al., 2017). Mental toughness is also a positive predictor of academic self-efficacy (Chen et al., 2020). Previous studies have shown that students with higher levels of mental toughness have higher levels of academic self-efficacy, and the two are positively correlated (Jiang et al., 2022; Liao, 2016). In summary, the physical and psychological exercises and challenges brought by physical exercise help to cultivate college students’ mental toughness so that they can better cope with the pressure and setbacks they face in the academic field, thus making it more likely that they will make positive progress in academic achievement, and this positive academic experience will in turn feed back into their perceptions of their own academic abilities and improve their academic self-efficacy. Based on the above analysis, this study proposes the hypothesis H3: Mental toughness mediates the relationship between physical exercise and college students’ academic self-efficacy.

### 2.4 Chain-mediated effects of future orientation and mental toughness

According to previous research, future orientation positively predicts psychological resilience (Li et al., 2015), which is an individual’s tendency and preference for the future (Mai, 2023). Psychological resilience is a reflection of an individual’s effective response and good adaptation in life, and is closely related to socio-emotional competence. College students with a positive future orientation often have clear plans and optimistic expectations for the future, and tend to regard short-term setbacks as a necessary process to achieve long-term goals (Liu et al., 2023), so that individuals are more likely to take the initiative to seek a variety of resources and opportunities to achieve their future goals, and will inevitably encounter a variety of difficulties and setbacks in the process. However, it is because of the positive beliefs about the future that they will regard these difficulties as a necessary path to their goals, thus stimulating their own psychological resilience to better cope with stress and setbacks. Based on the above analysis, this study proposes Hypothesis H4: Future orientation and psychological toughness play a chain mediating role in physical exercise and college students’ academic self-efficacy.

Combined with the above analysis of existing literature, it was found that there is a significant correlation between physical exercise, academic self-efficacy, future orientation and mental toughness and both, therefore, this study hypothesized that there is a chain mediating effect of future orientation and mental toughness in physical exercise and academic self-efficacy. Hypothesis H5 is proposed: future orientation and mental toughness have a chain-mediated effect between physical exercise and college students’ academic self-efficacy.

**FIGURE 1:**
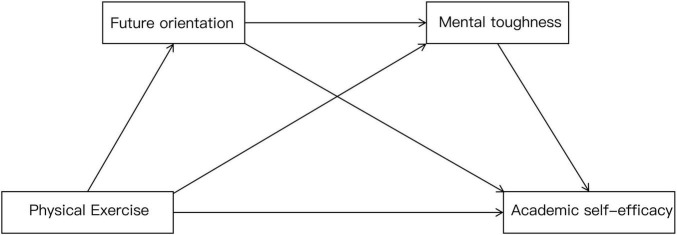
Hypothesized model diagram of physical exercise affecting academic self-efficacy.

As a result, the research hypothesis model was constructed as shown in Figure 1.

## 3 Research objects and methods

### 3.1 Research object

The research object of this paper is the effect of physical exercise on college students’ academic self-efficacy and the mediating role of future orientation and mental toughness. Separate measurement time in the research process for procedural control, in order to reduce the subject’s guessing about the purpose of the measurement. In order to reduce the interference of common method bias, reduce the memory effect of the subjects, and avoid systematic bias due to memory association, the questionnaires in this study were distributed in two sessions with a 2-week interval. The survey was conducted in several colleges and universities in Dalian City in December 2024, and stratified random sampling was used to draw samples from undergraduate college students stratified by gender, grade and major to ensure the representativeness of the samples. A total of 649 questionnaires were recovered, of which 25 students were excluded due to incomplete test information, yielding 624 valid subjects and a questionnaire validity rate of 96.15%. The demographics of the participants are detailed in the Supplementary Table.

### 3.2 Research instruments

#### 3.2.1 Physical Exercise Rating Scale (PARS-3)

The Physical exercise Rating Scale (PARS) revised by Liang Deqing et al. was used (Liang, 1994). It aims to assess the intensity, time, frequency and other indicators of the subjects’ participation in physical exercise in the past month. Each index was scored on a 5-point Likert scale (Likert scale). The subjects’ exercise was measured in terms of intensity, time, and frequency dimensions. Higher scores indicate a higher level of students’ participation in sports. The internal consistency Cronbach’s alpha coefficient of the scale in this study was 0.732, with good reliability and validity.

#### 3.2.2 Academic Self-Efficacy Scale

The academic self-efficacy questionnaire developed by Liang (2004) was used, including 22 questions in 2 dimensions: academic ability efficacy and academic behavior efficacy. The questionnaire was rated on a 5-point Likert scale from 1 “not at all” to 5 “completely,” with higher scores indicating higher academic self-efficacy. Among them, 7, 11, and 12 questions are reverse scoring questions. The Cronbach’s alpha coefficient for this questionnaire in this study was 0.932.

#### 3.2.3 Future orientation rating scale

The future orientation scale for adolescents compiled by Liu et al. (2011) et al. was used in this study. The scale contains 31 items and is mainly used to measure adolescents’ future orientation. The scale is rated on a 5-point scale, with 1 indicating complete non-conformity and 5 indicating complete conformity. The higher the score, the deeper the individual thinks about the future. The Cronbach’s alpha coefficient for this scale in this study was 0.928.

#### 3.2.4 Mental toughness rating scale

The mental toughness scale developed by Connor and Davidson and revised by Yu et al. (2007) was used in this study. A 5-level scoring was used. The higher the score, the higher the level of mental toughness. This scale has demonstrated excellent reliability and validity in the domestic college student population (Chen et al., 2021). In this study, the Cronbach’s alpha coefficient of this scale was 0.968.

### 3.3 Statistical methods

This study used SPSS 29.0 statistical software for data analysis. First, test reliability was tested using Cronbach’s alpha test and Harman’s one-way test was used to test for common method bias after data collection. Second, after importing the data into SPSS, demographic analysis was performed using descriptive statistics. Pearson correlation coefficients were used to analyze the correlations between physical exercise, academic self-efficacy, and future orientation with mental toughness. The study was analyzed by performing multiple regression analysis using Model 6 in the PROCESS macro program and evaluating the significance level of the mediating effect using Bootstrap test. In this study, the standardized beta coefficient (β) indicates the magnitude of change in the dependent variable for every one standard deviation change in the independent variable; ΔR^2^ represents the proportion of the variance in the dependent variable explained by the model, and ΔR^2^ represents the additional contribution of the added variable to the explained variance.

## 4 Results and analysis

### 4.1 Common method bias test

Due to the influence of the measurement environment, questionnaire guidance and context, the questionnaire survey to obtain data may have common method bias problems (Zhou and Long, 2004). The Harman one-way test was conducted on the measurement data, and the unrotated principal component analysis of physical exercise, academic self-efficacy, future orientation and mental toughness was conducted using SPSS 29.0, and the results showed that there were a total of 10 factors with eigenroots greater than 1, and the first factor explanation rate was 39.115%, which was less than the critical value of 40%, which indicated that the data of the present study did not have the problem of common method bias. Although Harman’s tests indicated that the results were at an acceptable level, measures such as anonymous surveys and randomization of the order of questions were taken to reduce common method bias.

### 4.2 Statistics and correlation analysis of the current status of physical exercise, academic self-efficacy, future orientation, and mental toughness among college students

#### 4.2.1 Demographics

Descriptive statistical analysis showed (Table 1) that there were 624 university students in the sample, of which 476 (76.3%) were male and 148 (23.7%) were female. This indicates that there are more male than female college students participating in the survey. In terms of grade distribution, freshmen had the most students with 276 students, accounting for 44.2%, sophomores had 197 students, accounting for 31.6%, juniors had 117 students, accounting for 18.8%, and seniors had 34 students, accounting for 5.4%. In terms of majors, there were 134 students in liberal arts, accounting for 21.5%, 369 students in science, accounting for 59.1%, and 121 students in arts and sports, accounting for 19.4%.

**TABLE 1:** Demographic characteristics of participants.

Causality	Form	Quantities	Percentage
Gender	Male student	476	76.30%
	Female student	148	23.70%
Grade	First-year university student	276	44.20%
	Second-year university student	197	31.60%
	Third-year university student	117	18.80%
	Fourth-year university student	34	5.40%

#### 4.2.2 Independent samples *t*-test

Independent samples *t*-test was used to analyze (Table 2), the differences in physical exercise, academic self-efficacy, future orientation, and mental toughness among college students of different genders. The results showed that male students were better than female students in physical exercise, academic self-efficacy, future orientation and mental toughness, with male and female students showing significant differences in physical exercise.

**TABLE 2:** Comparison of differences in variables among students of different genders.

	Gender	*N*	M ± SD	*t*	*p*
Physical exercise	Male	476	30.242 ± 25.860	5.76	<0.01
	Female	148	17.541 ± 22.618		
Academic self-efficacy	Male	476	3.614 ± 0.666	1.568	0.118
	Female	148	3.526 ± 0.570		
Future orientation	Male	476	3.388 ± 0.614	0.756	0.450
	Female	148	3.350 ± 0.509		
Mental toughness	Male	476	3.751 ± 0.715	0.895	0.371
	Female	148	3.692 ± 0.666		

#### 4.2.3 ANOVA one-way analysis

ANOVA one-way analysis (Table 3) was used to analyze the differences in physical exercise, academic self-efficacy, future orientation, and mental toughness among college students of different grades. LSD multiple comparisons were used to see if there were significant differences in physical exercise among the grades, and the results showed (Table 4) that freshmen and the other three grades showed significant differences in physical exercise.

**TABLE 3:** Comparison of differences in various variables among students of different grades.

Relevant variable	Grade	M ± SD	*F*	*P*
Physical exercise	1 (276)	31.080 ± 27.950	4.717	*P* < 0.05
	2 (197)	26.060 ± 23.276		
	3 (117)	22.368 ± 24.206		
	4 (34)	19.471 ± 19.776		
Academic self-efficacy	1 (276)	3.566 ± 0.674	1.131	0.336
	2 (197)	3.624 ± 0.640		
	3 (117)	3.559 ± 0.603		
	4 (34)	3.754 ± 0.569		
Future orientation	1 (276)	3.353 ± 0.613	0.402	0.752
	2 (197)	3.409 ± 0.594		
	3 (117)	3.379 ± 0.591		
	4(34)	3.421 ± 0.543		
Mental toughness	1 (276)	3.742 ± 0.698	0.645	0.586
	2 (197)	3.756 ± 0.737		
	3 (117)	3.832 ± 0.633		
	4 (34)	3.737 ± 0.703		

**TABLE 4:** Differential analysis of physical exercise of students in different grades.

Grade	First-year university student (31.080)	Second-year university student (26.060)	Third-year university student (22.368)
Fourth-year university student (19.471)	11.609^*,**^	6.590	2.897
Third-year university student (22.368)	8.712^*,**^	3.693	
Second-year university student (26.060)	5.019^*,**^		

*p < 0.05,

**p < 0.01.

#### 4.2.4 Pearson correlation analysis

Through Pearson correlation analysis of data on physical exercise, college students’ academic self-efficacy, future orientation and mental toughness (Table 5), it was found that there was a significant positive correlation between physical exercise and academic self-efficacy, future orientation and mental toughness, and there was also a significant positive correlation between future orientation and mental toughness and academic self-efficacy.

**TABLE 5:** Correlation analysis of physical exercise, academic self-efficacy, future orientation, and mental toughness.

	Physical exercise	Academic self-efficacy	Future orientation	Mental toughness
Physical exercise	1			
Academic self-efficacy	0.223**	1		
Future orientation	0.171**	0.605**	1	
Mental toughness	0.244**	0.670**	0.716**	1

Pearson correlation coefficient.

**p < 0.01.

### 4.3 Tests of mediating effects

As shown in Table 6, the chained mediation effect was tested using model 6 in the SPSS PROCESS macro program developed by Hayes. The results showed that: in the future orientation (M1) model, the standardized regression coefficient of physical exercise on future orientation was β = 0.181 (*p* < 0.001), indicating that for every 1 standard deviation increase in physical exercise, future orientation increased by 0.181 standard deviations. Physical exercise was a significant positive predictor of mental toughness (β = 0.127, *p* < 0.001), with physical exercise and future orientation together explaining 52.8% of the variance in mental toughness (*R*^2^ = 0.528) in the mental toughness (M2) model, and the three variables explaining a total of 48.8% of the variance in the academic self-efficacy (Y) model (*R*^2^ = 0.488). Future orientation was a significant positive predictor of mental toughness (β = 0.695, *p* < 0.001) and academic self-efficacy (β = 0.255, *p* < 0.001), and mental toughness was a significant positive predictor of academic self-efficacy (β = 0.471, *p* < 0.001). Including future orientation and mental toughness together in the structural equation, physical exercise had a significant positive predictive effect on college students’ academic self-efficacy (β = 0.231, *p* < 0.001).

**TABLE 6:** Regression analysis of the relationship of variables in the model.

Equation of regression		Overall fit index			Significance of regression coefficient		
Result variable	Variable of prediction	R	R2	F	β	*t*	*p*
Future orientation	Physical exercise	0.180	0.032	6.897	0.181	4.424	0.000^**,***^
	Gender				0.008	0.193	0.847
	Grade				0.057	1.416	0.157
Mental toughness	Physical exercise	0.727	0.528	173.062	0.127	4.369	0.000^**,***^
	Future orientation				0.695	24.748	0.000^**,***^
	Gender				0.104	0.367	0.714
	Grade				−0.008	−0.374	0.709
Academic self-efficacy	Physical exercise	0.698	0.488	117.581	0.066	2.158	0.031^*,**^
	Future orientation				0.255	6.17	0.000^**,***^
	Mental toughness				0.471	11.236	0.000^**,***^
	Gender				−0.022	−0.755	0.450
	Grade				0.046	1.59	0.112
Academic self-efficacy	Physical exercise	0.235	0.055	12.049	0.231	5.725	0.000^**,***^
	Gender				−0.013	−0.321	0.749
	Grade				0.074	1.884	0.060

*p < 0.05,

**p < 0.01,

***p < 0.001.

#### 4.3.1 Bootstrap mediation effect analysis results

This study focuses on the influence of physical exercise on college students’ academic self-efficacy, and the chain mediation model of physical exercise and academic self-efficacy was constructed and validated by introducing the two variables of future orientation and mental toughness. The results of the study show (Table 7) that the total effect value of physical exercise on academic self-efficacy is 0.0058, the direct effect value is 0.0017, the total indirect effect value is 0.0041, and the mediating effects of future orientation, mental toughness and the interaction between the two have reached the statistical significance level. The results showed that the chain mediation model of the research hypothesis was established and all four hypotheses were tested.

**TABLE 7:** Results of bootstrap mediated effects analysis.

Effect	Impact pathway	Effect value	BOOT SE	LLCL	ULCL	ΔR^2^	Proportion
Total effect		0.0058	0.001	0.0038	0.0078	0.488	100%
Direct effect	Direct path	0.0017	0.0008	0.0001	0.0032	0.055	29.31%
Total indirect effect		0.0041	0.0007	0.0027	0.0056	0.433	70.69%
Indirect effect	Ind1	0.0012	0.0004	0.0005	0.0019	0.015	20.69%
	Ind2	0.0015	0.0004	0.0007	0.0024	0.022	25.86%
	Ind3	0.0015	0.0004	0.0008	0.0023	0.031	25.86%

From the model, it can be seen that physical exercise predicts academic self-efficacy and that future orientation and mental toughness play an indirect mediating effect (three paths in total), with a total indirect effect value of 0.0041 and Bootstrap 95% confidence interval not containing 0 (LLCL = 0.0027, ULCL = 0.0056), which accounts for 70.69% of the total effect. The first mediating effect path: physical exercise → future orientation → academic self-efficacy (Path 1), with an indirect effect value of 0.0012, accounting for 20.69% of the total effect; the second path: physical exercise → mental toughness → academic self-efficacy (Path 2), with an indirect effect value of 0.0015, accounting for 25.86% of the total effect; and the third chain mediating effect path: Physical exercise → Future Orientation → Mental Toughness → Academic Self-Efficacy (Path 3), with an indirect effect value of 0.0015, accounting for 25.86% of the total effect, suggesting that the research hypothesis H4 is valid.

**FIGURE 2:**
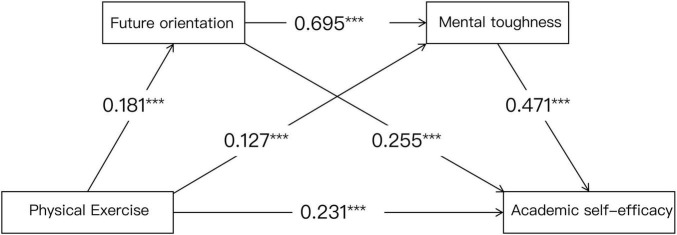
Pathways of physical exercise’s effect on academic self-efficacy. *** Indicates a significant difference.

Based on the above findings, the chain mediation model is shown in Figure 2.

## 5 Discussion

The present study explored the chain-mediated role of future orientation and mental toughness of physical exercise in the process of influencing college students’ academic self-efficacy, which is a positive exploration of physical exercise to enhance college students’ academic self-efficacy. From a theoretical perspective, this study not only enriches the influence mechanism of academic self-efficacy, especially analyzes the roles of future orientation and mental toughness, but also deepens the role of physical exercise in promoting college students’ mental health and academic performance; from a practical perspective, the study provides a concrete path for college students to carry out physical exercise, strengthen the cultivation of future orientation, improve mental toughness, and thus enhance academic self-efficacy; from a practical perspective, the study provides a concrete path for college students to carry out physical exercise, strengthen the cultivation of future orientation, improve mental toughness, and thus enhance academic self-efficacy. From a practical point of view, the study provides specific paths and theoretical support for college students to carry out physical exercise, strengthen the cultivation of future orientation, improve mental toughness, and thus enhance academic self-efficacy, which has strong practical guidance significance.

### 5.1 Physical exercise has a significant positive effect on college students’ academic self-efficacy

The results of the study show that physical exercise is correlated with academic self-efficacy, and physical exercise can significantly and positively predict academic self-efficacy, which is basically consistent with the results of existing studies (Guo and Wei, 2020). The direct effect of physical exercise on academic self-efficacy was significant (β = 0.231, *p* < 0.001), indicating that for every 1 standard deviation increase in physical exercise, academic self-efficacy increased by 0.231 standard deviations. The direct path independently explained 5.5% of the variance in academic self-efficacy (ΔR^2^ = 0.055), suggesting that physical exercise itself is an important driver of academic confidence. Male students demonstrated higher levels of physical exercise, but did not show a significant increase in academic self-efficacy. This may be due to biological differences between male and female students, with traditional gender role expectations tending to encourage males to demonstrate competitiveness through physical exercise, while female students may maintain academic levels through non-physical behaviors. Male students may view exercise as a stress release route rather than a means to directly promote academic confidence, while female students are more focused on balancing exercise with academic goals. This situation may result in male students being more actively engaged in physical exercise, but both genders are driven by similar educational goals in their academic self-perceptions, thus weakening the difference in perceived efficacy. The process of physical exercise requires students to focus (Kennelly and Toohey, 2014), overcome difficulties, and acquire specific skills and strategies. College students can enhance their self-efficacy through physical exercise (Chen, 2023), which enables them to demonstrate greater executive ability, a more optimistic mindset, and greater perseverance in the academic process (Qin, 2023). Therefore, we can visualize the effect of participating in physical exercise on college students’ academic self-efficacy.

### 5.2 The mediating effect of future orientation between physical exercise and college students’ academic self-efficacy

In this study, future orientation was found to be a mediating variable between physical exercise and academic self-efficacy, and H2 was verified. In the mediating path of future orientation, physical exercise indirectly enhanced academic self-efficacy (β = 0.255) by enhancing future orientation (β = 0.181), contributing 20.69% of the total effect. This pathway independently explained 1.5% of the variance in academic self-efficacy (ΔR^2^ = 0.015), suggesting that future orientation plays a unique role in goal setting and planning, but its explanatory power was lower than the direct effect (ΔR^2^ = 0.055). In terms of the separate mediating role of future orientation, physical exercise can bring positive effects to all levels of the body, making individuals more relaxed in soothing adverse emotions such as anxiety and depression. Acquiring a more positive future orientation (Kahana et al., 2005; Sun et al., 2024). The stronger the academic self-efficacy of college students, the higher the level of their educational expectations and planning (Wang, 2020), and the more confident they are in their learning abilities and behaviors, the more they will think about and explore their future education, which is in line with the results of previous studies. This result is consistent with previous studies. Future orientation helps college students to develop healthier and better living habits, and has a positive value in the acquisition of academic self-efficacy and the development of life plans (Zhang, 2022). Therefore, the higher the level of future orientation, the higher the academic self-efficacy of college students.

### 5.3 The mediating effect of mental toughness between physical exercise and college students’ academic self-efficacy

Physical exercise can positively predict mental toughness, and there is a significant mediating effect of mental toughness in physical exercise and academic self-efficacy, which is basically consistent with the existing studies (Kong, 2018; Wang and Bu, 2023; Zhou and Zhou, 2022). In the mediating path of mental toughness, physical exercise indirectly enhanced academic self-efficacy (β = 0.471) by increasing mental toughness (β = 0.127), contributing 25.86% of the total effect. This pathway independently explained 2.2% of the variance (ΔR^2^ = 0.022), highlighting the central value of mental toughness in coping with academic stress, with higher explanatory power than future orientation (ΔR^2^ = 0.015). During physical exercise, college students are exposed to stress related to learning motor skills, challenges originating from competitors, and resulting frustration. These conditions may adversely affect the psychological health of college students in a short period of time, but when they successfully overcome these problems by their own efforts or with the assistance of peers and teachers, they will be able to build a more resilient psychological state and improve their problem-solving ability, so that they can effectively cope with and adapt to stressful situations in their lives, and maintain an optimistic and positive attitude toward their lives and emotional state. Whether in the midst of adversity or under less stressful situations, mental toughness allows individuals to remain optimistic and energetic in their lives, and ensures and enhances their sense of wellbeing. Strengthening the mental toughness of college students can effectively improve their academic self-efficacy (Li, 2023). In conclusion, physical exercise can improve the mental toughness of college students, and mental toughness significantly affects the formation and development of college students’ academic self-efficacy, and mental toughness plays a mediating effect in this process.

### 5.4 Chain mediating effects of future orientation and mental toughness in physical exercise and college students’ academic self-efficacy

In exploring the relationship between physical exercise on academic self-efficacy, this study found that future orientation and mental toughness could constitute a significant chain-mediated effect. The chain mediation model showed that future orientation and mental toughness together explained 70.69% of the total effect (indirect effect value = 0.0041), with future orientation alone contributing 20.69% (β = 0.181 →β = 0.255, ΔR^2^ = 0.015) and mental toughness contributing 25.86% (β = 0.127 →β = 0.471, ΔR^2^ = 0.022), while the chained path of both further contributed 25.86% (β = 0.181 →β = 0.695 →β = 0.471, ΔR^2^ = 0.031). This suggests that physical exercise not only directly enhances academic self-efficacy through enhancing future orientation (ΔR^2^ = 0.031 → 0.488), but also indirectly strengthens this process through the development of mental toughness, highlighting the synergistic role of multidimensional psychological mechanisms in academic development. Validating existing research, future orientation positively predicts mental toughness. College students with high future orientation have higher expectations for the future, stronger motivation and intention, and are able to choose learning styles that are more suitable for them, enhance their interest in learning, gain more positive academic emotions during the learning process, deploy their limited cognitive resources into learning, promote the formation of their own learning perseverance, enhance their psychological toughness, and continue to increase the amount of investment in learning (Wang et al., 2022). The chain mediator proposed in this study is an integration of research on the relationship between future orientation and mental toughness and academic self-efficacy, which is conducive to a more comprehensive understanding of the internal mechanism of the impact of physical exercise on college students’ academic self-efficacy, and is of great significance in promoting the reform of school sports.

## 6 Conclusion

First, physical exercise, future orientation, mental toughness and academic self-efficacy are significantly correlated. Second, physical exercise can significantly and positively predict college students’ academic self-efficacy, which is an important intervening variable affecting college students’ academic development. Third, future orientation and mental toughness not only have a simple intermediary role in the process of physical exercise affecting college students’ academic self-efficacy, but also can affect college students’ academic self-efficacy through the chain of future orientation and mental toughness, and this kind of indirect intermediary effect has an important impact on the development of college students’ academic self-efficacy.

### 6.1 Research limitations and future directions

This study investigates the inner mechanism of the role of physical exercise and academic self-efficacy to provide some theoretical basis for related research on improving college students’ academic level. Although the data and model fit well, there are still some shortcomings: (1) This study adopts a cross-sectional design, which is not deep enough to argue the causal relationship of the variables, and in the future, a longitudinal tracking design can be adopted to further explore the relationship between the variables. (2) This study is only based on the model of chain mediation to illustrate the relationship among the four, and other alternative models can be further considered in the future to explore the relationship between these factors and learning input. (3) This study selected college students in Liaoning Dalian universities as the research sample, and did not involve college students from other regions and other groups. Future research should further expand the scope of the study and select a wider range of research subjects to test the applicability of the findings. (4) The survey object of this study is only for undergraduates in colleges and universities, while whether secondary school students and primary school students are equally suitable for this research model, and whether the pathway mechanism of exercising to improve academics is the same as that of college students can be further explored in future studies.

## Data Availability

The original contributions presented in the study are included in the article/Supplementary material, further inquiries can be directed to the corresponding author.
